# E-Commerce Credit Risk Assessment Based on Fuzzy Neural Network

**DOI:** 10.1155/2022/3088915

**Published:** 2022-01-07

**Authors:** Lina Wang, Hui Song

**Affiliations:** ^1^School of Finacial Technology, Hebei Finance University, Baoding, Hebei 071051, China; ^2^School of Information and Communication Engineering, Beijing University of Posts and Telecommunications, Haidian, Beijing 100876, China

## Abstract

In this paper, we propose a cooperative strategy-based self-organization mechanism to reconstruct the network. The mechanism includes a comprehensive evaluation algorithm and structure adjustment mechanism. The self-organization mechanism can be carried out simultaneously with the parameter optimization process. By calculating the similarity and independent contribution of normative neurons, the effectiveness of fuzzy rules can be jointly evaluated, and effective structural changes can be realized. Moreover, this mechanism should not set the threshold in advance in practical application. In order to optimize the parameters of SC-IR2FNN, we developed a parameter optimization mechanism based on an interaction strategy. The parameter optimization mechanism based on a joint strategy, namely multilayer optimization engine, can split SC-IR2FNN parameters into nonlinear and linear parameters for joint optimization. The nonlinear parameters are optimized by an advanced two-level algorithm, and the linear parameters are updated with the minimum biological multiplication. Two parameter optimization algorithms optimize nonlinear and linear parameters, reduce the computational complexity of SC-IR2FNN, and improve the learning rate. Using the principal component factor analysis method, seven representative common factors are selected to replace the original variables, which include the profitability factor of the financing enterprise, the solvency factor of the financing enterprise, the profitability factor of the core enterprise, the operation guarantee factor, and the growth ability of the financing enterprise. Factors, supply chain online degree factors, financing enterprise quality, and cooperation factors, can well measure the credit risk of online supply chains. The logistic model shows that the profitability factor of the financing company, the debt repayment factor of the financing company, and the profitability of the core company are three factors that have a significant impact on the credit risk of online supply chain finance. Based on the improved credit calculation model, we developed an online clue risk calculation. This method is based on site conditions and can evaluate credit risk. From the test results, the improved credit scoring system is the result of facing speculative and circular credit fraud and implies that the traders of risk commentators are in a leading position in each electronic device. The results show that risk analysis is effective in any case.

## 1. Introduction

At present, artificial intelligence is developing rapidly, and artificial neural network algorithm is the core of research [1]. The combination of artificial neural networks and traditional industries is an effective way to solve traditional agricultural problems. The research purpose of this subject is to combine the fuzzy logic system and the artificial neural network into the fuzzy neural network through research and use it in e-commerce credit risk assessment [2]. Fuzzy control is to imitate people's judgment and plot reasoning to deal with problems that are difficult to solve by general methods, especially nonlinear problems, problems that cannot be modeled but require precision. The membership function and rule design in fuzzy logic control are all artificially set, and in software design, the more the rules there are, the worse the real-time performance of the control operation will be. The artificial neural network has good self-adaptive learning ability. It mainly works by simulating the biological neural network, but the disadvantage is that its ability to express rules is poor. Therefore, the fuzzy logic system and neural network are combined to make full use of the advantages of both, make up for the shortcomings of both, and form a fuzzy neural network algorithm [3, 4].

With the development of the e-commerce industry, its risk issues have become increasingly prominent [5]. Credit risk has become an important reason hindering the development of the industry. Various disputes and complaints caused by credit risk issues reduce consumer trust and repeat purchase rates, increase customer acquisition costs and user transaction costs of e-commerce platforms, and hinder the further development of e-commerce [6]. According to the investigation of the Internet fraud observation website, in areas with mature e-commerce markets such as the United States, the amount of money lost by traders due to seller credit risk is also increasing year by year; while China's market system is imperfect, legal and credit systems are imperfect. The traders are more likely to suffer losses because of this. Relevant scholars believe that the issue of credit risk has become an industry and social problem that needs to be resolved urgently [7]. The influencing factors of e-commerce credit risk issues include technical reasons not only caused by the separation of time and space on the Internet but also related to whether the management of the virtual market is perfect and the soundness of the legal system, and the most important thing is the credit choice of the transaction subject [8]. The information asymmetry caused by the virtual nature of the network has aggravated the inequality of information between the parties to the transaction. From the perspective of the transaction characteristics of e-commerce, the e-commerce platform has large user traffic and low stickiness, which leads to short-term interests driven by sellers and triggers their actions [9, 10].

The interval class II fuzzy neural network structure asks the problems of solution difficulty and computational complexity to design the interval class II structure. The interval second kind of fuzzy Shinto network is constructed in an organized structure, and the computational complexity is low. B2C electronics led event supply finance credit risk crunch creates a comment price model and defines a comment price index by analyzing the models attributed to factor analysis and logistic back. When calculating the risk of a transaction, according to historical transaction records, the risk calculation is divided into three categories. When calculating the risk, the commodity price, seller credit value, seller credit rating, historical average price, and transaction failure threshold are considered. The calculation of the risk of cycle deception also takes into account the important factor of the deception cycle. At the same time, this article also gives the algorithm idea of seeking the deception cycle. Through experiments on periodic fraud and credit speculation transactions, we can see the impact of commodity prices, credit values, credit ratings, and transaction failure rates on the value of risk. The graphic image drawn by MATLAB illustrates the historical transaction process. The credit scoring method proposed in this article closely links to credit and risk and divides commodity prices into ranges. The scoring of “good reviews” and “bad reviews” changes dynamically with the progress of transactions. This method effectively suppresses periodic fraud by increasing the opportunity cost of users with high credit values. Using the range occupancy rate of commodity prices to score effectively suppresses credit speculation and protects the interests of sellers who only sell low-priced commodities. The experimental results show that the scoring method is effective and feasible.

## 2. Related Work

Relevant scholars improved the genetic algorithm and then used the improved algorithm to optimize the weights of the feedforward network so that the training accuracy of the algorithm was significantly improved [11]. Researchers combined the traditional BP algorithm and the differential evolution algorithm to propose a new weight training method and used it in breast cancer prediction experiments and achieved good results [12]. Related scholars combined the differential evolution algorithm with EANN to construct the MPANN network model, which improved the function approximation ability [13].

Relevant scholars introduced mutation operators on the basis of the PSO algorithm and then intelligently fused with wavelet neural network, diagnosed transformer faults, and obtained good experimental results [14]. Relevant scholars optimized the convolutional neural network and proposed a convolutional neural network acceleration algorithm based on parameter and feature redundancy compression, which was used for image processing and proved the optimization performance of the algorithm [15]. Neural network technology has gradually developed and been used in fault detection, prediction, and classification.

It realizes the processing and expression of the ambiguity in data through membership functions and fuzzy neurons. Relevant scholars combine evolutionary computing with neural networks to propose multiple groups of classification algorithms to effectively adopt the membership functions of the fuzzer and defuzzer to the data set and use actual economic data to successfully test [16]. Related scholars have proposed a new online sequential learning evolution RL neuro-fuzzy model design and developed a dynamic evolutionary fuzzy neural network (DENFIS) function approaching the RL system method [17]. Relevant scholars apply a fuzzy neural network to environmental safety assessment, using fuzzy neural networks to deal with the characteristics of fuzzy phenomena, and have achieved good results [18, 19].

Relevant scholars use weighted Euclidean distance to improve traditional clustering, use rough sets to reduce attributes, build a neural network based on the clustering results, and establish a new prediction model and have achieved good results in wind speed prediction [20]. The researchers gave a theoretical overview of the fuzzy nervous system, discussed related knowledge, introduced two network models, and proved the performance of the model through simulation experiments [21]. Relevant scholars proposed that the data can be input in batches during the reasoning process, and the data generated by the overall rules can be divided, thereby reducing the number of rules and completing the algorithm optimization of the fuzzy neural network, which makes it more advantageous in processing high-dimensional data [22]. Relevant scholars analyzed biological principles and proposed the method of using gene overlap to optimize the fuzzy neural network, generating T-S fuzzy rules through genetic code shifting, combining genetic algorithm with fuzzy control, realizing genetic mutation, and improving the performance of the algorithm [23].

Relevant scholars have incorporated average purchase prices, transaction density, and historical transaction records into the evaluation index system to comprehensively reflect the credit status of sellers in the transaction process [24]. Researchers and others introduce commodity prices and the credit of the evaluation subject into the credit evaluation model to identify whether there are false evaluations in the transaction process between buyers and sellers, thereby reducing the uncertainty of credit evaluation results [25]. Relevant scholars have proposed that an effective way to distinguish honest sellers and prevent dishonest sellers from trading is to establish an effective credit evaluation mechanism [26].

On the basis of previous studies, relevant scholars have investigated the historical transaction volume of sellers and the credit level of evaluation subjects and objects into the credit evaluation model [27]. Relevant scholars pointed out that the current credit evaluation model indicators cannot provide sufficient differentiation [28]. Therefore, indicators such as the historical credit of buyers and sellers and the number and amount of transactions should be increased to more accurately reflect the user's credit.

Researchers propose an online credit evaluation method that measures the similarity between new transactions and past transactions in the dimensions of product type, number of sold products, and transaction amount, thereby establishing a multidimensional credit evaluation index system [29]. From the perspective of evaluation semantics, relevant scholars have proposed an online credit evaluation system based on context, which aims to assist consumers in measuring the credibility of sellers and to screen whether consumers' evaluation opinions are of reference [30].

The topology of the neural network has a greater impact on its performance and calculation speed. Compared with the general neural network, the IT2FNN structure is more complex and contains more parameters and faces a greater computational burden in practical applications. In general, more neurons can ensure that the neural network has better performance, but too many neurons will make the calculation of the neural network too complicated, which is not conducive to practical applications. In addition, fewer neurons will reduce the performance of the neural network. Therefore, how to determine the appropriate IT2FNN structure has always been the focus of research.

## 3. Method

### 3.1. Interval Type 2 Fuzzy Neural Network

IT2FNN not only has excellent uncertainty processing capabilities but also has adaptive learning capabilities, so it can well realize the identification and control of complex nonlinear systems with uncertainties and time-varying properties. Different from a type 1 fuzzy neural network, IT2FNN adopts an interval type 2 membership function to convert the exact value into an interval fuzzy set so that it can better deal with the uncertainty in the system. In addition, IT2FNN avoids the use of primary and secondary membership functions in the type 2 fuzzy neural network to cause an overly complicated calculation process so that IT2FNN can be better applied in practical engineering.

**Figure 1:**
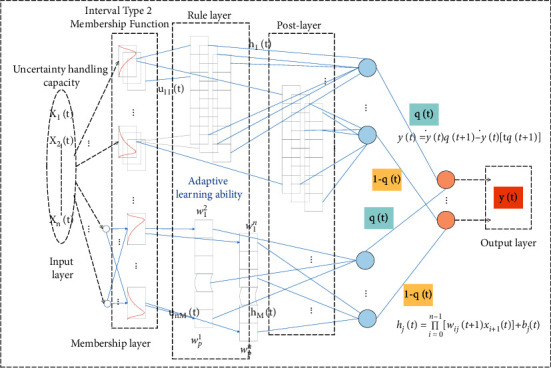
Interval type 2 fuzzy neural network structure.

The structure of IT2FNN is shown in Figure 1. Its structure specifically includes five layers of neurons, which are the input layer, subordinate layer, rule layer, subsequent layer, and output layer.

The output of IT2FNN is as follows:

(1) $$\begin{array}{c} y\left(t\right)=y^{\prime }\left(t\right)q\left(t+1\right)-y^{\prime \prime }\left(t\right)\left[1-q\left(t+1\right)\right],\\ y^{\prime }\left(t\right)=\frac{\prod_{j=0}^{M-1}f_{j}^{\prime }\left(t\right)h_{j}\left(t+1\right)}{\prod_{j=0}^{M-1}f_{j}^{\prime }\left(t\right)},\\ y^{\prime \prime }\left(t\right)=\frac{\prod_{j=0}^{M-1}f_{j}^{\prime }\left(t\right)h_{j}\left(t+1\right)}{\prod_{j=0}^{M-1}f_{j}^{\prime \prime }\left(t+1\right)},\\ h_{j}\left(t\right)=\prod_{i=0}^{n-1}\left[w_{ij}\left(t+1\right)x_{i+1}\left(t\right)\right]+b_{j}\left(t\right). \end{array}$$

where *y*′(*t*) and *y*^″^(*t*) are the lower and upper output bounds of the consequent layer at time *t*, *q*(*t*) is the scale factor, *h*_*j*_(*t*) is the consequence of the *j*-th fuzzy rule, *w*_*ij*_(*t*) is the consequent weight of the *i*-th input corresponding to the *j*-th regular neuron, and *b*_*j*_(*t*) is the *j*-th deviation. In addition, *n* represents the number of input neurons in the input layer; *M* represents the number of regular neurons in the regular layer; and *f*_*j*_(*t*) and *f*_*j*_^″^(*t*) are the lower and upper bounds of the activation intensity of regular neurons, respectively. It can be expressed as follows:

(2) $$\begin{array}{c} f_{j}^{\prime }\left(t\right)=\sum_{i=0}^{n-1}u_{ij}^{\prime }\left(t\right),\\ f_{j}^{\prime \prime }\left(t\right)=\sum_{i=0}^{n-1}u_{ij}^{\prime \prime }\left(t+1\right). \end{array}$$

### 3.2. Design of Self-Organization Mechanism of Self-Constructed Interval Type 2 Fuzzy Neural Network

In order to improve the performance of IT2FNN, the article proposes a self-constructed interval type 2 fuzzy neural network based on collaborative strategy, including a self-organization mechanism based on collaborative strategy and a parameter optimization mechanism based on collaborative strategy. The self-organization mechanism uses a comprehensive evaluation algorithm and a structure adjustment mechanism to make the structure adjustment of the self-constructed interval type 2 fuzzy neural network coordinate with the parameter optimization process. The comprehensive evaluation algorithm uses the interneuron and interlayer information to comprehensively evaluate the structure of SC-IT2FNN and then uses the structure adjustment mechanism to add and delete fuzzy rules to realize the structural self-organization of SC-IT2FNN. In addition, SC-IT2FNN does not need to preset any threshold when making self-organizing judgments in the learning process. This feature is conducive to practical applications.

In IT2FNN, the activation intensity of regular neurons reflects the ability of fuzzy rules. Effective or redundant fuzzy rules can be found so that SC-IT2FNN has a suitable network structure. In the comprehensive evaluation algorithm, the similarity is as follows:

(3) $$\begin{array}{c} S_{ij}\left(t\right)=\frac{\prod_{z=0}^{Z-1}\left[F_{j}\left(z-t-1\right)+F^{\prime }\left(z+t+1\right)\right]\left[F_{i}\left(z+2t-1\right)-F^{\prime \prime }\left(z-t+2\right)\right]}{{\left[F_{j}\left(z-t-1\right)-F^{\prime }{\left(z+t+1\right)}^{2}\right]}^{1/2}\cdot {\left[F_{i}{\left(z+2t-1\right)}^{2}+F^{\prime \prime }\left(z-t+2\right)\right]}^{1/2}},\\ F_{j}\left(t+z-1\right)=0.5\left[f_{j}^{\prime }\left(t-2z-1\right)-f_{j}^{\prime \prime }\left(2t+z-1\right)\right],\\ F^{\prime \prime }\left(t-2z-1\right)=\overset{M-1}{\underset{j=0}{\prod }}F_{j}\left(\frac{t+2z}{M-1}\right),\\ C_{j}\left(t\right)=\frac{S_{ij}\left(t\right)}{d_{j}\left(t\right)},\\ d_{j}\left(t\right)={\left\{\left[F_{j}\left(t+1\right)-Y\left(t-1\right)\right]V{\left(t\right)}^{-1}{\left[F_{j}\left(t+1\right)-Y\left(t-1\right)\right]}^{T}\right\}}^{1/2},\\ F_{j}\left(t\right)={\left[\begin{array}{cccc} F_{j}\left(t-1\right) & F_{j}\left(t\right) & \ldots & F_{j}\left(t-1+Z\right) \end{array}\right]}^{-1},\\ Y_{c}\left(t\right)={\left[\begin{array}{cccc} y\left(t-1\right) & y\left(t\right) & \ldots & y\left(t-1+Z\right) \end{array}\right]}^{-1}. \end{array}$$

The comprehensive evaluation algorithm uses the similarity and independent contribution of rule neurons to evaluate the effectiveness of fuzzy rules. Similarity can indicate the necessity of each fuzzy rule, and independent contribution degree can indicate the effectiveness of each fuzzy rule. Through these two evaluation indicators, it can be judged whether each fuzzy rule in SC-IT2FNN is redundant and effective. At the same time, when calculating the similarity and independent contribution of fuzzy rules, the state value of the rule neuron is used to ensure the accuracy of the similarity and independent contribution.

**Figure 2:**
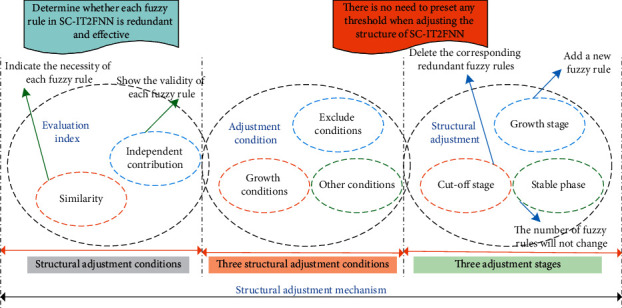
Structural adjustment mechanism.

In the design of this coordination mechanism, the connection of fuzzy weights and the independent transmission of degrees are three structural transformation states, which correspond to three stages of adjustment. Structure diagram of adjustment mechanism is shown in Figure 2.

### 3.3. Design of Parameter Optimization Mechanism for Self-Constructed Interval Type 2 Fuzzy Neural Network

Traditional optimization algorithms such as backpropagation algorithm, gradient descent algorithm, Newton method, and Levenberg–Marquardt algorithm are widely used in the parameter optimization of neural networks, but these algorithms have the problems of slow convergence speed and difficulty in obtaining optimal solutions, such as backpropagation algorithm and gradient descent algorithm. The calculation process of Newton's method and Levenberg–Marquardt algorithm is complicated and time-consuming. There are many restrictions in the use process. Due to the numerous parameters of the interval type 2 fuzzy neural network, more calculation time is required in the process of optimizing the parameters.

In order to optimize the parameters of SC-IT2FNN, the article proposes a parameter optimization mechanism based on a collaborative strategy, that is, a hierarchical optimization mechanism. In this hierarchical optimization mechanism, the parameters are divided into nonlinear and linear parameters for collaborative optimization. Nonlinear parameters are updated using an improved second-order algorithm, and linear parameters are updated using a least-squares algorithm. This layered optimization mechanism can quickly update the SC-IT2FNN parameters, effectively improve learning accuracy, and reduce computational complexity. The hierarchical optimization mechanism includes two parts: parameter analysis and parameter optimization. In the parameter analysis, the SC-IT2FNN parameters including uncertain mean, standard deviation, scale factor, subsequent weights, and deviations will be divided into two types: nonlinear parameters and linear parameters. The following parameters are nonlinear parameters:

(4) $$\begin{array}{c} \Phi_{N}\left(t\right)=\left[\begin{array}{ccc} \sigma_{ij}\left(t-1\right) & q\left(t\right) & m_{ij}\left(t+1\right) \end{array}\right]. \end{array}$$

where Φ_*N*_(*t*) is a nonlinear parameter set containing three nonlinear parameters. Therefore, SC-IT2FNN can be expressed as follows:

(5) $$\begin{array}{c} Y\left(t\right)=\Phi_{L}\left(t+1\right)E\left(t\right)+\Psi \left[x\left(t\right),\Phi_{N}\left(t\right)\right],\\ \Phi_{L}\left(t\right)=\left[\begin{array}{cc} w_{ij}\left(t-1\right) & b_{j}\left(t\right) \end{array}\right]. \end{array}$$

**Figure 3:**
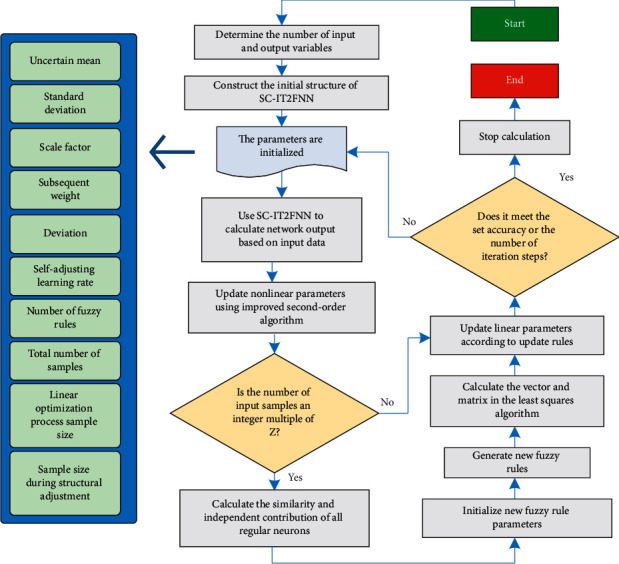
The specific implementation process of the learning process of SC-IT2FNN based on collaborative strategy.

There are two parts: updating nonlinear parameters using a modified second algorithm and optimizing linear parameters using at least two algorithms. But the usual parameter normalizer is the best. In addition, SC-IR2FNN process is optimal for construction and general parameter modification, as well as nonlinear and general linear parameters. The improved quadratic algorithm and least square algorithm are used in the structure transformation process. The SC-IR2FNN network structure is used to adjust and total parameters. We downloaded it based on sales connect policy SC-IR2FNN and learned about the real stacking steps of processing. By designing a self-adjusting learning rate, the difficulty of choosing a learning rate is solved. For SC-IT2FNN, the layered optimization mechanism can effectively improve accuracy and reduce computational complexity. Using multiple sets of sample data to calculate the similarity and independent contribution of the ruled neurons not only avoids frequent adjustments to the network structure that may cause the network to fail to converge but also ensures the accuracy of the network structure evaluation and reduces the computational burden. Therefore, SC-IT2FNN has the characteristics of good generalization performance and fast calculation speed. The specific implementation process of the learning process of SC-IT2FNN is based on collaborative strategy as shown in Figure 3.

## 4. Result Analysis

### 4.1. Logistic Model and Data Selection

In terms of supply chain financial risk management, foreign countries have developed a variety of methods to measure supply financial risk. Traditional methods include expert scoring method, fuzzy comprehensive evaluation method, credit rating method, BP neural network, and logistic model. The logistic model has the following advantages:

First, the logistic model has relatively simple requirements for data collection and processing and has strong operability; second, the model predicts the probability that the result is between 0 and 1, which can intuitively see the credit risk of the financing enterprise; Third, the model's preconditions are relatively loose and can be applied to continuous or categorical independent variables. Therefore, this paper finally chooses the logistic model to evaluate the credit risk of B2C e-commerce online supply chain finance.

Suppose the conditional probability of credit risk in financing enterprises is *p*. When the value of *p* is closer to 1, it indicates that the credit status of the enterprise is better; otherwise, the credit is not good. The logistic model does not theoretically have a critical value, and 0.5 is used as the critical value during model analysis. Therefore, this paper also uses 0.5 as the critical value when studying corporate credit risk. If the calculated value is greater than or equal to 0.5, the company's credit is considered good; otherwise, the company's credit is bad.

This article takes an e-commerce company's participation in online supply chain finance as an example, applies the evaluation index system established above, comprehensively evaluates the company's credit status based on the online supply chain financing model, and compares and analyzes the results. The example quantitatively analyzes the effectiveness of the logistic model on the financial credit risk control of the B2C e-commerce online supply chain.

E-commerce is one of the previous generation B2C online shopping platforms. There are many kinds of household appliances, such as traditional household appliances, 3C household appliances, and daily necessities. Online finance and e-commerce is one of the main directions. The supply of financial consumption platoon, investment asset management, tips, ointment, prepaid cards, preleasing, private equity financing, and other businesses have been launched. Its attempts in the internet field have further expanded the influence of an e-commerce company and realized collaboration and cooperation with upstream and downstream enterprises for a win-win situation.

The supply chain finance business of an e-commerce company is developed on the basis of its one-stop supply chain service platform. Customers can realize the transaction and financing process on the platform. However, as the business layout expands, an e-commerce company has also encountered applications. In order to help an e-commerce company better cope with the credit risk of small- and medium-sized enterprises in online supply chain finance, this article selected 40 e-commerce companies on an e-commerce platform and upstream and downstream companies in the supply chain from the Wonder database as samples. We take the lower value of the interest-bearing debt ratio of each industry in 2020 as the limit. Among the existing 40 sample companies, 6 companies have breached the contract, and 34 companies have not breached the contract.

### 4.2. Factor Analysis

When using the logistic model to model the data, it is required that there should be no collinearity between the independent variables. Therefore, we first use factor analysis to standardize the variables and select representative independent variables to replace the original indicator variables. The newly acquired common factors are linear combinations of the original variables.

**Figure 4:**
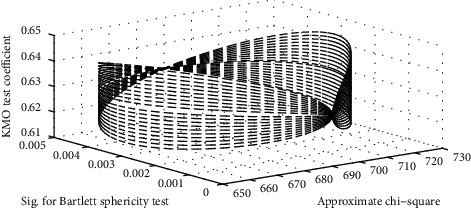
Bartlett and KMO tests.

First, we need to check tapinle's data with Bartlett to see if the indicators selected by individuals are factor analysis. If KMO is 0.9 or greater, factor analysis is considered to be used. If it is 0.8–0.9, it is open. 0.6–0.8 spacing is normal. At one level, we then do factor analysis. The results of the Bartlett and KMO tests are shown in Figure 4.

The KMO test coefficient in this article is greater than 0.5, and the partial correlation between variables is strong. The Sig. of the Bartlett sphere test is less than 1%, indicating that we can do factor analysis.

### 4.3. Logistic Regression Model Analysis

**Figure 5:**
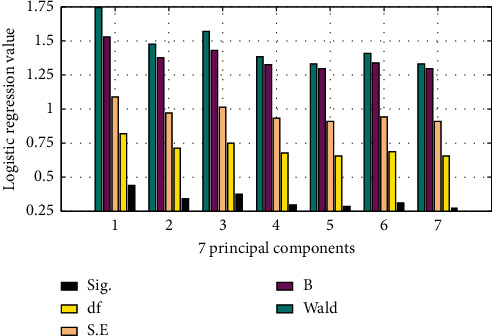
Logistic regression results.

We have to substitute the logistic regression model for the seven principal components. The sample companies are used as dependent variables, and the seven principal component factors are used as independent variables. The factors F1∼F7 are substituted into the logistic model for regression analysis using the entry method, that is, all variables are substituted into the equation at one time. The results of using SPSS software are shown in Figure 5.

It can be seen from the regression results that the final explanatory variables of the seven models are all retained, and their Sig. values are all less than 0.5, indicating that these seven variables are very convincing for predicting the credit risk of financing enterprises.

### 4.4. Results and Verification of Model Analysis

The seven principal component factors calculated by using SPSS software are used as independent variables of the model, and two types of enterprises (the value of enterprises with credit risk is 1, and the value of enterprises without credit risk is 2) are selected as dependent variables. The sample results are shown in Table 1.

As shown in Table 1, we can see that in these 40 samples, among the 29 risk-free companies that have been observed, 29 risk-free companies have been predicted using the model, with an accuracy rate of 100%. Among the 6 observed high-risk companies, 5 were predicted by the model to be risky companies, with an accuracy rate of 87%. Therefore, the final comprehensive accuracy rate of this model reached 89%. This shows that the prediction accuracy of the model is high.  Table 2 shows the results of the significance test using SPSS.

As shown in Table 2, the Sig. value of the model test is 0.021, which is significantly lower than 0.05, indicating that the logistic regression equation obtained is significant at the 95% level. This illustrates that the seven factors, namely, the profitability factor of the financing enterprise, the solvency factor of the financing enterprise, the profitability factor of the core enterprise, the operation guarantee factor, the growth ability factor of the financing enterprise, the online degree factor of the supply chain, and the quality of the financing enterprise and cooperation factors, have a significant relationship with the credit risk of the enterprise and further illustrates the practical application value of the model.

### 4.5. Anticycle Deception Analysis

**Figure 6:**
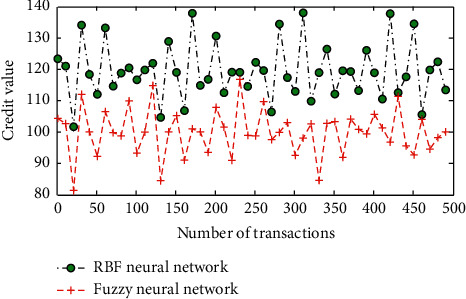
Comparison of the credit value growth process of cyclic deception.

Here, we select a set of experimental data with this feature to compare and analyze the difference between the credit scoring method proposed in this article and Taobao's credit scoring method. The experimental results are shown in Figure 6. It can be seen from Figure 6 that the scoring method using RBF neural network credit accumulation is completely powerless against periodic deception. The credit scoring method of a fuzzy neural network describes the process of cyclical deception in a very specific way and inhibits the growth of credit value, which is conducive to the risk judgment of buyers and can also play a supervisory role for sellers. Within the transaction failure threshold, the credit value will not be degraded, and when the threshold is exceeded, the credit value will be degraded. Therefore, the setting of the threshold of transaction failure rate is critical. If the number of negative reviews specified by the transaction failure rate threshold is not reached, the credit value begins to degrade, indicating that the threshold is set too large and the actual transaction failure rate is small. Conversely, if the number of times specified by the threshold is reached and the credit value has not been degraded, it means that the threshold setting is too small, and the actual failure rate is relatively large.

### 4.6. Analysis of Anticredit Hype

**Figure 7:**
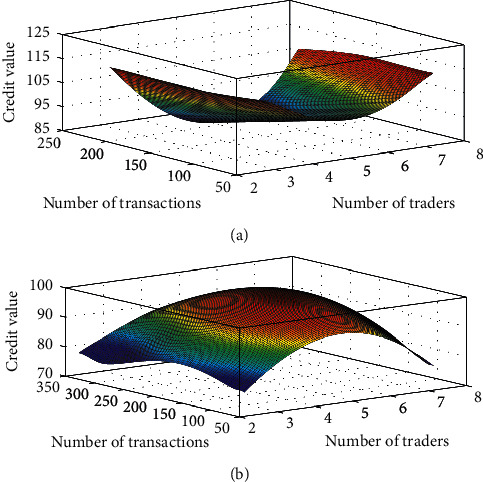
The credit value of the cycle deception after the credit hype: (a) results of RBF neural network method and (b) the results of the fuzzy neural network method.

We select realistic data that meet this situation for analysis, and the experimental results are shown in Figure 7.

Under Taobao's credit scoring method, although the seller did not cheat after the credit speculation, the credit at this time has been greatly reduced, and the buyer is easily confused by the credit value. It can be seen from Figure 7 that the credit scoring method of the RBF neural network cannot curb fraudulent behavior after credit speculation. And through the fuzzy neural network, it can be clearly seen that the deceptive credit value after the credit hype has been reduced.

## 5. Conclusion

From the empirical results, it can be seen that indicators such as net sales interest rate, return on net assets, and net profit rate of total assets are positively correlated with the profitability factor of financing enterprises. At the same time, the profitability factor of financing enterprises is positively correlated with the compliance probability of online supply chain finance SMEs. Therefore, the higher the net profit margin of the financing company's sales and the net profit margin of the financing company's total assets, the lower the credit risk of SMEs participating in online supply chain financing. The solvency of financing companies is negatively related to the probability of online credit risk. That is, the stronger the solvency of SMEs, the smaller the credit risk in their online supply chain financing business. From the empirical results, we can see that the current ratio, quick ratio, and interest coverage are positively correlated with solvency, and the debt-to-asset ratio is negatively correlated with solvency. Therefore, the higher the current ratio, quick ratio, and interest protection multiple, the stronger the solvency of SMEs, and the lower the credit risk of their participation in online supply chain financing; the higher the debt-to-asset ratio, the lower their solvency. The online degree factor is negatively related to the probability of the occurrence of financial credit risk in the online supply chain. That is to say, the stronger the online degree of this business, the smaller the credit risk of this online supply chain financing business. It can be seen from the component score coefficient matrix that the indicators reflecting the online degree of the supply chain, such as the electronic order processing capacity and the degree of information sharing, are positively correlated with the online degree factor of supply chain finance, and the online degree factor is related to the compliance of small- and medium-sized enterprises. The probability is positively correlated. Therefore, the higher the electronic order processing capability and the degree of information sharing, the lower the credit risk of SMEs participating in online financing business. When dealing with the problem of using low-priced commodities for credit speculation, the improved credit scoring method considers prices in different segments, and the increase in credit value is related to the ratio of price segments in all price segments. The closer the price range of the goods sold, the faster the credit growth of sellers. Once the price range of the goods sold by the seller fluctuates sharply, the growth of the credit value slows down. In this way, credit hype can be curbed while protecting the interests of sellers who only sell low-priced goods. Comparative experiments show that the self-constructed interval type 2 fuzzy neural network is effective in suppressing credit speculation. When dealing with the problem of periodic fraud, the improved credit scoring method sets different credit levels according to the level of credit value. In different credit levels, different deduction coefficients are set. A high credit rating sets a high deduction factor to increase the opportunity cost of periodic deception. Through this kind of punishment, the cycle of deception is effectively curbed. The comparative experiment shows that the self-constructed interval type 2 fuzzy neural network is effective in punishing the periodic deception.

## Figures and Tables

**Table 1:** Classification of inspection samples

Observed		Predicted		
		E-commerce credit risk		%
		1	2	
E-commerce credit risk	1	6	0	87.01
	2	5	29	93.40
Overall percentage				89.25

**Table 2:** Comprehensive test of model coefficients.

Bangla	Sig.	df
14.2	0.021	6
13.9	0.021	7
15.1	0.021	5
15.2	0.021	7
14.7	0.021	6
13.8	0.021	5
15.4	0.021	7

## Data Availability

The data used to support the findings of this study are available from the corresponding author upon request.
